# Government policy attention to aging and mental health among middle aged and older adults: A text-based analysis of chinese municipal reports

**DOI:** 10.1371/journal.pone.0337347

**Published:** 2025-12-03

**Authors:** Chong Zhuo, Ling Dai, Yuyang Deng

**Affiliations:** 1 School of Population and Health, Renmin University of China, Beijing, China,; 2 School of Social Development and Public Policy, Fudan University, Shanghai, China,; 3 College of Business, Shanghai University of Finance and Economics, Shanghai, China; 4 CIB Research Company Limited, Shanghai, China; Nanjing Audit University, CHINA

## Abstract

**Objective:**

Population aging has intensified mental health challenges among middle-aged and older adults. This study examines how government attention to aging issues affects mental health outcomes in this demographic.

**Methods:**

Using data from the China Health and Retirement Longitudinal Study (CHARLS) and municipal government work reports collected between 2011 and 2020, we constructed a government attention index. We employed fixed effects models to analyze the impact on depressive symptoms among middle-aged and older adults, exploring underlying mechanisms and population heterogeneity.

**Results:**

Higher government attention significantly reduces depressive symptoms among middle-aged and older adults, with stronger effects observed in men and rural populations. Four key mechanisms drive these improvements: environmental enhancement, expanded social welfare, increased adult children visitation, and improved family financial status. Government attention also promotes participation in social and cultural activities but shows no significant impact on adult children’s economic support or sleep duration.

**Conclusion:**

Government attention to aging issues substantially improves mental health outcomes among middle-aged and older adults. Policymakers should strengthen responses to create more supportive social environments for this growing demographic.

## 1 Introduction

China’s rapidly aging population has made the health issues of middle-age and elderly people increasingly prominent [1–5]. According to the seventh national census, the population aged 65 and above has reached nearly 200 million, accounting for 13.5% of the total population. This demographic shift has fundamentally altered China’s population structure and created numerous social challenges, particularly heightened mental health risks [6–8]. Mental health issues such as depression and anxiety are increasingly common among the elderly [9–13], with depression detection rates reaching approximately 20.6% among the elderly population from 2018 to 2022.

Traditionally, families provided primary emotional support for elderly individuals. However, rapid urbanization and population mobility have created widespread empty-nest and lone-living elderly populations in China [14,15]. The weakening of traditional support networks has intensified mental health pressures among the elderly [16,17], creating an urgent need for alternative support mechanisms.

China has responded at the policy level through initiatives like the Healthy China Initiative (2019–2030), which aims to comprehensively improve population mental health through medical services, health education, and intervention mechanisms [18]. However, limited resource allocation and incomplete support systems mean that older adults’ mental health receives less attention than their material security. While government attention to aging issues is not a direct intervention measure, it may guide social resource allocation and public opinion through policy signals, creating more favorable environments for elderly mental health [19,20]. For example, the 2024 Government Work Report frequently addressed aging-related issues across social security, public services, healthcare, and technology sectors, suggesting that government positions on public agendas may enhance societal awareness of elderly needs, thereby driving mental health resource allocation.

Existing research has primarily examined how government policies affect the economic [21,22] and medical conditions [23,24] of elderly populations, with limited attention to mechanisms through which policies enhance psychological well-being via non-material pathways such as policy promotion and social advocacy. Furthermore, the interactive relationship between social support and family support requires deeper examination [25,26]. Some studies have overlooked the potentials’ substitution effect’ between these support systems, where improvements in social welfare may reduce family members’ motivation to provide care, leaving elderly individuals emotionally isolated despite economic security [27]. This phenomenon is particularly relevant in Chinese family culture [28].

This paper extends the analysis to middle-aged and older adults aged 45 and above, encompassing those transitioning from working-age to retirement. This population faces distinct mental health risks and must cope with career transitions and retirement adaptation pressures, making their inclusion analytically important. The study addresses three key questions: First, can government attention to aging issues improve mental health among middle-aged and older adults through direct policy implementation or signaling effects? Second, does social support partially replace family support, thereby affecting its psychological buffering effect? Third, can the government enhance mental health policy effectiveness through indirect interventions, specifically through environmental enhancement, social welfare expansion, family support reinforcement, and economic security improvement?

To address these questions, this paper constructs a text-based indicator measuring government attention levels within the macro context of an aging society. Using data from the China Health and Retirement Longitudinal Study (CHARLS) collected between 2011 and 2020, we assess mental health levels among middle-aged and older adults. Fixed-effects models and instrumental variable methods are employed to identify causal effects of government attention while exploring the underlying mechanisms, including policy support, signal transmission, social governance, and family support.

This paper makes three marginal contributions: First, it introduces the government attention to aging indicator, expanding research perspectives on older adults’ mental health. Existing research has primarily examined how government policies affect the economic [21,22] and medical conditions [23,24] of elderly populations, with limited attention to mechanisms through which policies enhance psychological well-being via non-material pathways. This paper focuses on examining the mechanisms through which governmental attention to ageing influences older adults’ mental health via non-material pathways, such as policy advocacy and social promotion, broadening the research perspective on mental health issues among older adults.

Secondly, when examining the mechanisms through which government influences older adults’ mental health via material and non-material pathways, this study proposes the hypothesis that social support and family support may exhibit a “substitution effect”. Although previous literature has discussed the relationship between social support and family support [25], it has overlooked the potential substitution effect between these two forms of support, that is to say, enhanced social welfare may diminish family members’ motivation to provide care and reduce financial support for the elderly [27]. This study empirically examines the relationship between social and family support, finding that increased governmental attention to ageing issues significantly elevates the level of social welfare received by middle-aged and elderly individuals, yet has no significant impact on the financial support provided by their children. This outcome validates the partial substitutability between socioeconomic support and familial financial support.

Third, it constructs quantitative indicators based on government reports, enhancing measurement scientific rigor and policy explanatory power while providing theoretical foundations and empirical support for relevant policy design. This paper adopts the measurement methods for government attention proposed by Oster et al [29]. We manually collected government work reports from prefecture-level cities across China from 2011 to 2020. Using word segmentation technology, we screened 56 keywords related to aging, including aging, elderly care, retirement, and silver economy. We extracted the number of characters in keywords and their sentences, calculating their proportion of total characters in government work reports. This approach effectively reflects government attention to aging issues while preserving semantic and syntactic meaning of original texts, reducing information loss from simple word frequency counting.

## 2 Theoretical explanation

As the global population ages rapidly, governments increasingly focus on aging-related issues [30,31]. Previous research has primarily emphasized physical health [32,33], particularly the importance of improving social security systems [34]. The promotion of urban and rural resident medical insurance has significantly increased medical treatment rates among rural residents [35], while pilot programs for long-term care insurance have effectively reduced the burden on families caring for disabled elderly individuals [36]. However, elderly mental health has emerged as a critical public health issue [37–39]. In response, governments have implemented various measures, including strengthening mental health services and building elderly-friendly communities, with particular focus on rural and empty-nest elderly populations. By improving living environments and strengthening social support networks, governments have significantly enhanced elderly mental health outcomes [40,41].

Existing research primarily focuses on two dimensions: social support and family support. Social support, encompassing material and emotional assistance obtained through social relationships, positively impacts elderly psychological well-being [42,43]. Family support includes financial assistance, daily care, and emotional communication. Within traditional filial piety culture, intergenerational support has improved elderly living conditions while enhancing their sense of belonging and security [44,45].

**Fig 1:**
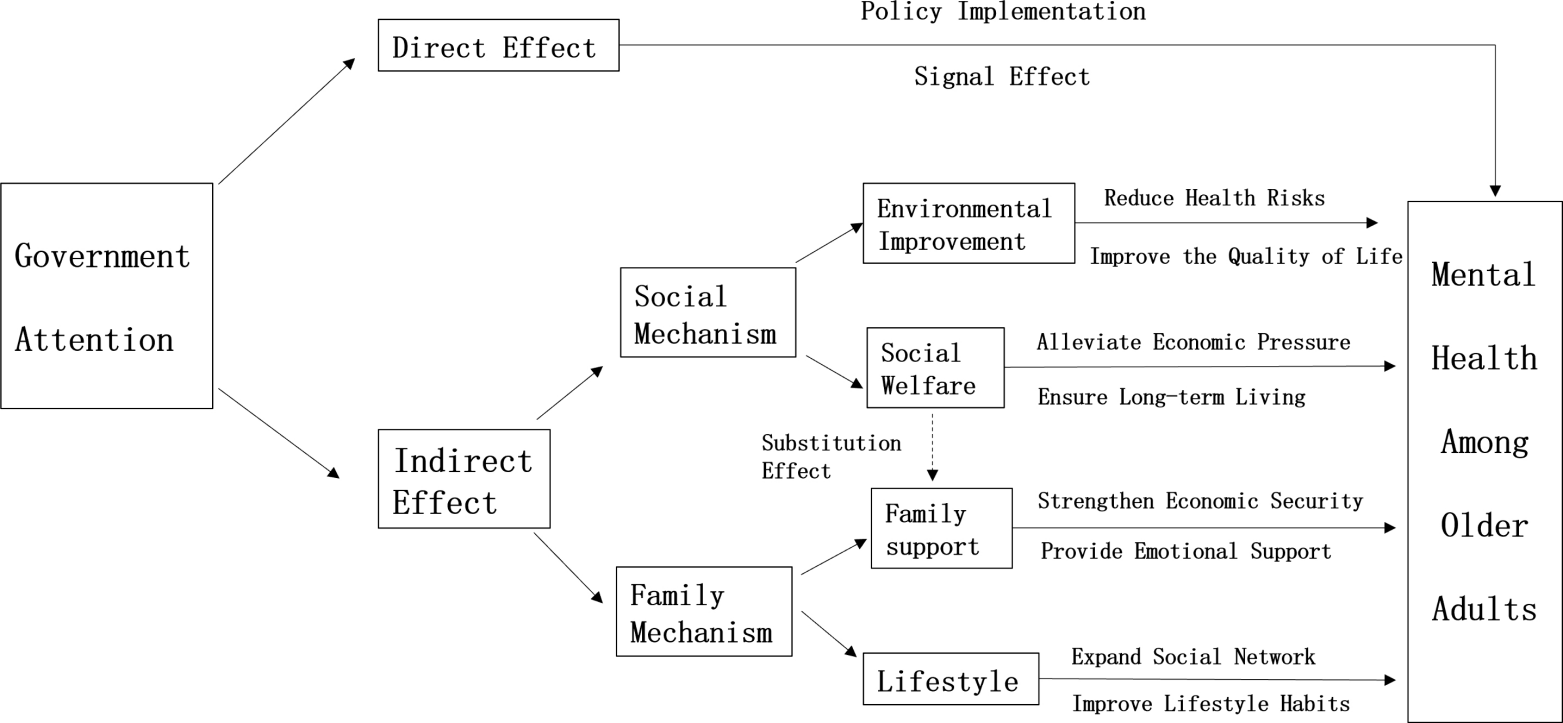
Mechanism of government attention to aging on mental health of middle-aged and elderly adults. *Notes:* This figure illustrates the mechanism through which government attention to aging affects the mental health of middle-aged and elderly individuals. Figure 1 was compiled by the author.

Nevertheless, social transformation and population mobility have led to family miniaturization, with adult children and parents living separately becoming the norm, thereby weakening traditional support networks. While existing research provides theoretical support for elderly mental health, systematic research on government attention to mental health remains limited, and the interactive mechanisms between government policies, social support, and family support remain relatively understudied. This paper proposes a mechanism framework to explore how local government policies influence middle-aged and elderly individual’s mental health through both direct and indirect effects (Fig 1).

### 2.1 The direct effect of increased government attention to aging

Government attention to aging issues directly impacts middle-aged and elderly mental health through two channels: policy implementation effects and signal effects.

#### Policy implementation effects.

Government improves social support systems for middle-aged and elderly populations by formulating and implementing aging-related policies [46]. Regarding economic support, social welfare and subsidies alleviate living burdens, reducing anxiety and insecurity [47]. In health security, improving public healthcare systems and strengthening basic medical insurance alleviate illness-related concerns [48]. Through social services, providing community support facilities expands social spaces and reduces loneliness [49].

#### Signal effects.

Government sends caring for the elderly social signals through policy documents and public statements. When middle-aged and elderly individuals recognize that government highly values their group’s interests, their psychological sense of security increases, improving social status recognition and life confidence.

### 2.2 The indirect effect of increased government attention to aging

Government attention to aging indirectly improves middle-aged and elderly mental health through four mechanisms:

#### Environmental improvement mechanism.

Government indirectly improves middle-aged and elderly living conditions by strengthening environmental governance and pollution control. Enhanced environmental quality reduces health threats, improves physical condition, reduces anxiety and psychological stress, and increases life satisfaction and security [50].

#### Social welfare mechanism.

Government increases social welfare spending, alleviating eco-nomic pressure by improving social security and medical subsidies. This reduces uncertainty in health expenditure by expanding medical insurance coverage and establishes comprehensive long-term welfare systems to enhance life security and autonomy [51].

#### Family support mechanism.

Government promotes elderly respect culture [52], improves community services, and encourages adult children to actively participate in elderly care. Government support reduces economic burdens on adult children, allowing them to devote more energy to emotional companionship, promoting family harmony, and indirectly improving elderly mental states.

#### Lifestyle mechanism.

Government builds community activity centers, promotes cultural and sports activities, and conducts health education to guide middle-aged and elderly individuals toward healthy lifestyles [53]. Social interaction enhances social connections and belonging, cultural and sports activities help relieve stress and improve mood, and regular schedules help control emotional fluctuations [54].

## 3 Methods and data

### 3.1 Empirical design

Based on the theoretical analysis above, this paper constructs a multidimensional fixed effects model as the benchmark econometric model:


$$depression_{i,t}=\beta_{\text{0}}+\beta_{\text{1}}{\text{attention}}_{i,t}+\beta_{\text{2}}{\text{control}}_{i,t}+fe_{year}+fe_{family}+fe_{city}+\varepsilon_{i,t}$$ (1)

In equation 1 i and *t* represent the sample individuals and observation years, respectively. $depression_{i,t}$ is the dependent variable, representing the degree of depression among middle-aged and elderly people; ${\text{attention}}_{i,t}$ is the independent variable, representing the degree of government attention to the aging issues; ${\text{control}}_{i,t}$ denotes control variables at the household and city levels; $\beta_{\text{0}}$, $\beta_{\text{1}}$, $\beta_{\text{2}}$ are the parameters to be estimated.

$fe_{year},\;fe_{family},fe_{city}$ represent fixed effects for year, household, and city. The purpose is to control for the impact of non-time-varying factors at the household and city levels on the degree of depression among middle-aged and elderly people, while also controlling for the impact of time-varying factors such as macroeconomics that do not change with individuals, thereby reducing the potential impact of omitted variables. $\varepsilon_{i,t}$ is the standard error clustered at the household level, as there may be some unobserved factors at the household level that affect all members of the household. By clustering at the household level, the impact of these factors on the estimation results can be controlled to a certain extent.

### 3.2 Data description

The sample data for this study primarily originates from municipal government work reports, CHARLS, the China Urban Statistical Yearbook, and the CNRDS database. Since CHARLS data collection began in 2011, surveys have been conducted biennially. This study selects individuals aged 45 and above as the research population. Through data screening, cleaning, and matching, we obtained five waves of panel data covering 115 prefecture-level cities, 18,342 households, and 35,637 individuals (35,637 represents the total sample size of all observations used in the regression analysis) across China for the years 2011, 2013, 2015, 2018, and 2020. Descriptive statistics for each variable are presented in Table 1.

**Table 1:** Descriptive Statistics.

Variable	Description	Mean	Std. Dev.
**Individual-Level Variables**			
Depression level	Depression scale score (higher = more severe)	7.786	6.036
Government attention	Aging-related terms in government reports	3.235	2.199
Age	Age in years	60.75	9.591
Education years	Years of formal education	5.989	4.367
Marital status		1.747	1.480
Gender	1 = Male, 2 = Female	1.502	0.500
Household registration		1.303	0.568
Healthcare expenditure	ln(household medical spending)	5.293	3.762
Number of children	Total number of children	2.112	1.634
Social welfare	Total transfer income received (yuan)	44.445	127.583
Child visit frequency	10 = high intensity, 1 = low intensity	5.272	2.209
Financial support	Annual financial support from children	3913.4	11310.565
Urban/rural	0 = Rural, 1 = Urban	0.401	0.490
Social interaction	Frequency of social activities (binary)	0.413	0.492
Recreational activities	Frequency of recreational activities (binary)	0.248	0.432
Sleep duration	Daily sleep hours	6.208	1.841
**Household-Level Variables**			
Household savings rate	Savings/ total financial assets (%)	4.324	5.777
Household debt ratio	Debt/ total financial assets (%)	14.986	852.094
**City-Level Variables**			
SO₂ emission intensity	ln(SO₂ emissions/ GDP)	34.378	69.836
Log(GDP)	Natural log of GDP	7.726	0.934
Log(fiscal expenditure)	Natural log of fiscal expenditure	14.216	1.158
Log(physicians)	Natural log of licensed physicians	9.355	0.725
Log(hospital beds)	Natural log of hospital beds	10.036	0.652
Log(hospitals)	Natural log of hospital count	5.096	0.698
Log(hospitals)	Natural log of education spending	13.529	0.757
Wastewater emission	ln(wastewater emissions/ GDP)	3.718	4.813
Soot/dust emission	ln(soot-dust emissions/ GDP)	24.831	129.666

*Notes*: The data presented are primarily sourced from five waves of CHARLS data (2011, 2013, 2015, 2018, 2020), government work reports, and the China City Statistical Yearbook.

The CHARLS is a high-quality micro-dataset representative of households and individuals aged 45 and above in China. The CHARLS national baseline survey was conducted in 2011, covering approximately 17,000 individuals from about 10,000 households. We primarily utilize data from five waves spanning 2011, 2013, 2015, 2018, and 2020 for our analysis. This study adhered to the ethical principles outlined in the 1964 Helsinki Declaration and its subsequent amendments. The data used in this study were retrieved from the CHARLS. This survey was endorsed by the Biomedical Ethics Committee of Peking University (NO.IRB 00001052–11015). All participants in the survey signed or marked (if illiterate) the informed consent forms. All methods were carried out in accordance with relevant guidelines and regulations.

#### Step 1: Individual-household data matching and cleaning.

We matched individual questionnaires with household questionnaires using household identification codes to create individual-household level datasets for each wave. For example, for the 2020 CHARLS dataset, we matched individual and household questionnaires and retained 13,423 valid individual samples after excluding observations with severe missing data. These samples originated from 105 prefecture-level cities and 5,519 different households. We applied the same procedure to the 2018, 2015, 2013, and 2011 datasets, ultimately constructing a panel dataset covering 18,342 households with a total of 35,638 individual sample observations from 2011 to 2020.

#### Step 2: Multi-level data integration.

We matched the prefecture-level city administrative codes of CHARLS sample locations with government work reports and city statistical yearbook data at the prefecture level. Government work reports were sourced from official websites of prefecture-level city governments, primarily summarizing major achievements from the past year and establishing work priorities for the coming year. This process created a three-level dataset (individual-household-regional) designed to examine the impact of macro-level government attention to aging on micro-level individuals.

### 3.3 Independent variable: Government attention to aging

Developing a scientifically sound government attention index helps comprehensively and accurately assess macro policy impacts on middle-aged and older adults’ mental health. Existing studies often extract term frequency or occurrence rates related to relevant policy areas from government work reports and other guiding documents as indicators of policy intensity or attention. While this method reflects policy emphasis in specific areas, relying solely on word frequency proportions may overlook context, semantics, and sentence meaning in policy texts, leading to measurement biases.

Therefore, this paper adopts the measurement methods for government attention proposed by Oster et al [29]. We manually collected government work reports from prefecture-level cities across China from 2011 to 2020. Using word segmentation technology, we screened 56 keywords related to aging, including aging, elderly care, retirement, and silver economy. We extracted the number of characters in keywords and their sentences, calculating their proportion of total characters in government work reports. This approach effectively reflects government attention to aging issues while preserving semantic and syntactic meaning of original texts, reducing information loss from simple word frequency counting. Additionally, to avoid duplicate counting errors, sentences containing multiple keywords are counted only once in statistics. Since local government work reports are typically released at the beginning of the year while actual government activities span the entire year, this indicator has strong exogeneity, largely avoiding endogeneity caused by reverse causality and enhancing the reliability of empirical analysis.

### 3.4 Dependent variable: Degree of depression

The dependent variable is depression severity among middle-aged and older adults, calculated based on the depression scale (CES-D) in the CHARLS data collected from 2011 to 2020. The scale includes 10 items designed to assess respondents’ mental health status, such as I get annoyed over small things and I have diﬀiculty concentrating on tasks. Each item is categorized into four levels based on occurrence frequency: never (<1 day), not very often (1–2 days), sometimes or half the time (3–4 days), and most of the time (5–7 days). Depression scores are calculated according to standard criteria, with the four levels scoring 0, 1, 2, and 3 points respectively. Items 5 (I am hopeful about the future) and 8 (I am happy) are reverse-scored, resulting in total scores ranging from 0 to 30 points.

### 3.5 Other variables

This paper uses basic characteristic variables at the household or individual level as control variables, including age, years of education, marital status, gender, household registration, healthcare expenditure, and number of children. Additionally, we select socioeconomic characteristic variables at the city level as control variables, including sulfur dioxide emission intensity to measure environmental quality, GDP and fiscal expenditure to measure economic development levels, and the number of hospitals, licensed or assistant physicians, and hospital beds to measure public service levels.

According to the theoretical analysis, mechanisms through which government attention affects middle-aged and elderly mental health include environmental improvement, social welfare, family support, and lifestyle. We select relevant variables to measure these aspects: Environmental improvement: We use urban wastewater and particulate matter emission intensity to measure water pollution and air pollution in cities. Social welfare: We use total transfer payments received by respondents in CHARLS data as a measure. Family support: We measure family care using annual economic support provided by adult children to elderly individuals and the frequency of adult children visiting elderly parents from CHARLS data. For asset allocation, we calculate family savings rates and debt ratios. Lifestyle: This includes frequency of elderly participation in social activities, cultural and sports activities, and sleep duration.

The data processing for the variables (Table 1) is as follows: **Individual-level variables:** (1) Age: Calculated by subtracting the respondent’s birth year from the survey year. (2) Years of education: Assigned based on the respondent’s educational attainment level. When the individual education level is coded as 1–11, the corresponding years of education are 0, 3, 3, 6, 9, 12, 16, 15, 16, 19, and 22, respectively. (3) Gender: Coded as 1 for male respondents and 2 for female respondents. (4) Social interaction: Measured by the frequency of social activities. (5) Entertainment activities: Measured by the frequency of entertainment activities. (6) Sleep duration: Measured by daily sleep hours. (7) Medical expenditure: Represented by the logarithm of household medical expenses. (8) Social welfare: Measured by the total amount of transfer payments received. (9) Frequency of children visiting parents: First, the original data was reverse-coded using a 1–10 scale, where higher values indicate more frequent parental visits by children. Second, for families with multiple children, the frequency values were summed and averaged to obtain the mean frequency of children visiting parents. (10) Economic support: Measured by the annual financial support pro-vided by adult children to their parents. (11) Urban-rural classification: Represented by a binary variable, where 0 indicates rural areas and 1 indicates urban areas. **Household-level variables:** (1) Household savings rate: Calculated as the ratio of household savings to financial assets. (2) Household debt ratio: Calculated as the ratio of household debt to financial assets.

## 4 Research results

### 4.1 Baseline regression results

To examine the impact of government attention to aging issues on the mental health of middle-aged and elderly people, a fixed-effects model is used for benchmark regression analysis. Column (1) controls only for basic variables at the individual level, and the results show a significant negative correlation between government attention to aging issues and the degree of depression among middle-aged and elderly people. This indicates that, without controlling for other potential con-founding factors, local governments’ attention to aging issues helps alleviate depression symptoms among middle-aged and elderly people.

In columns (2) to (4), the model sequentially introduces year, household, and city fixed effects to control for the impact of time trends, unobservable factors at the household level, and inter-city heterogeneity on the estimation results. Regardless of which fixed effects are controlled for, the coefficient for government attention remains negative and statistically significant, indicating that this effect is robust. Notably, after incorporating household and city fixed effects, the coefficient for government attention further expands, suggesting that the positive impact of government policy attention on the mental health of middle-aged and older adults remains valid under stricter identification conditions, and may even be strengthened.

In summary, government attention to aging issues is significantly associated with lower levels of depression among middle-aged and older adults, and this relationship remains robust after controlling for potential confounding factors.

### 4.2 Analysis of heterogeneity in middle-aged and older adults

Heterogeneity analysis indicates that the impact of government attention to aging issues on the degree of depression among middle-aged and older people varies significantly across gender and living environments (Table 2).

**Table 2:** Baseline regression results.

	(1) Depression	(2) Depression	(3) Depression	(4) Depression
Government Attention	−0.034** (0.014)	−0.025* (0.015)	−0.041** (0.020)	−0.041** (0.020)
Control Variables	Yes	Yes	Yes	Yes
Year FE	No	Yes	Yes	Yes
Household FE	No	Yes	Yes	Yes
City FE	No	No	Yes	Yes
Observations	35,637	35,637	35,637	35,637
Adjusted R-squared	0.101	0.197	0.405	0.401

*Notes*: Numbers in parentheses below coefficients in column (1) are standard errors; Columns (2)-(4) report household-clustered standard errors. * *p <* 0.1, ** *p <* 0.05, *** *p <* 0.01.

**Fig 2:**
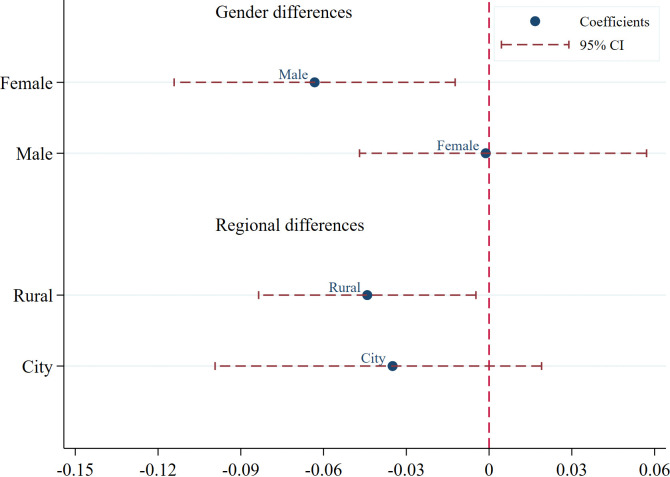
Heterogeneity analysis results. *Notes:* This figure presents the heterogeneous effects of government attention to aging on elderly mental health across gender and urban-rural classifications. The x-axis shows point estimates and 95% confidence intervals. Data are primarily sourced from the CHARLS data collected from 2011 to 2020, and government work reports. Figure 2 was compiled based on CHARLS data.

In terms of gender, the mitigating effect of government attention on depressive symptoms is significant among men but not among women. This difference may stem from differences in social support mechanisms: men are less likely to express their emotions in traditional culture, and the institutional and emotional support conveyed by government policies have a greater buffering effect on their psychological state. Women’s mental health, however, may be more influenced by factors such as family relationships, social role identity, and social support. Relying solely on government policy support is not sufficient to significantly improve their mental state (Fig 2).

In terms of place of residence, government attention has a significant mitigating effect on the degree of depression among rural middle-aged and elderly people, but no significant effect on urban populations. Possible explanations include: (1) Differences in public resources allocation: Rural area generally lag behind in social services and infrastructure. Government attention to aging issues and the implementation of corresponding policies can effectively compensate for the shortage of public resources in rural area, providing critical support for rural middle-aged and elderly people. In contrast, urban residents already have a relatively well-developed social security system, and marginal policy attention has a relatively limited impact on their mental health. (2) Differences in the causes of psychological stress: Urban middle-aged and elderly people may face more complex psychological stressors, such as the fast pace of urban life, weakened social connections, rising living costs, and feelings of loneliness brought about by urbanisation. Addressing these issues often requires comprehensive, multi-level, and cross-departmental interventions, and single-policy attention alone is unlikely to achieve substantial improvements (Fig 2).

### 4.3 Environmental improvement mechanism

To further explore how government attention affects the mental health of middle-aged and elderly people through environmental improvement mechanisms, this paper selects urban wastewater discharge intensity and smoke and dust emission intensity as key indicators of environmental quality. The regression results shown in columns (1) to (2) of Table 3 indicate that, increased government attention to aging issues significantly reduces the emission levels of these two types of pollutants, thereby improving environmental quality. These results indicate that while paying attention to the aging issues, the government has strengthened its efforts to control water and air pollution.

**Table 3:** Mechanism Analysis: Environmental Improvement, Social Welfare, and Family Support.

	Environmental Improvement		Social Welfare and Family Support		
	Wastewater (1)	Soot/Dust (2)	Social (3)	Financial (4)	Child Visit (5)
Government Attention	−0.112*** (0.007)	−0.924*** (0.043)	1.574*** (0.441)	4.617 (48.529)	0.014** (0.006)
Control Variables	Yes	Yes	Yes	Yes	Yes
Year FE	Yes	Yes	Yes	Yes	Yes
Household FE	Yes	Yes	Yes	Yes	Yes
City FE	Yes	Yes	Yes	Yes	Yes
Observations	22,244	20,515	35,637	35,637	35,637
Adjusted R-squared	0.830	0.994	0.197	0.313	0.350

*Notes*: All estimates control for survey year fixed effects, household fixed effects, and city fixed effects. * *p <* 0.1, ** *p <* 0.05, *** *p <* 0.01.

Water pollution poses a direct threat to the health of middle-aged and elderly people, particularly in terms of drinking water safety and living environments. With increased government attention, more resources have been allocated to wastewater treatment and water quality improvement, effectively reducing health risks associated with water pollution. Similarly, air pollution, especially particulate matter, poses a significant threat to the fragile respiratory systems of middle-aged and elderly people. Government policies have played a key role in reducing air pollutant emissions, thereby lowering the likelihood of middle-aged and elderly people developing respiratory diseases.

In summary, this mechanism ultimately helps to improve the overall health of middle-aged and elderly people and alleviate their feelings of depression [55–57]. Government attention indirectly improves the mental health of middle-aged and elderly people through environmental improvements.

### 4.4 Social welfare and family support

The results in columns (3) to (5) of Table 3 reveal the mechanisms of social welfare and family support in the process of government attention influencing the degree of depression among middle-aged and elderly people. The study finds that increased government attention to aging issues significantly increases the level of social welfare received by middle-aged and older adults and the frequency of visits from their adult children.

In terms of social welfare, this paper measures it by the total amount of transfer payments received by respondents. Higher total transfer payments indicate better social welfare for respondents. On one hand, this can meet the production and living needs of respondents and their families, raise their standard of living, and reduce their concerns about production and daily life, thereby alleviating depressive symptoms. On the other hand, higher total transfer payments enable respondents to better address physical and mental health conditions, lessen their suffering, and thus mitigate depressive symptoms. Therefore, enhanced social welfare effectively alleviates depressive symptoms among respondents [56,58,59].

In terms of family support, this study measures it through the annual financial assistance provided by children to their elderly parents and the frequency of visits made by children to their elderly parents. Although the government’s level of attention to ageing issues does not significantly influence the financial support provided by children, it does significantly increase the frequency of visits made by children to their elderly parents. Increased frequency of visits from children provides considerable psychological comfort to the elderly, significantly reducing their feelings of loneliness and thereby alleviating depressive symptoms. Therefore, enhanced family support effectively mitigates depressive symptoms among respondents [60–62].

However, it did not significantly affect the economic support provided by adult children. This result may be attributed to the following three factors:

(1) Substitution effect of economic support: As the government pays increasing attention to the issue of aging, social welfare such as pensions and medical insurance continues to improve. Middle-aged and elderly people can obtain more stable economic security through these public resources, thereby reducing their dependence on financial assistance from their adult children. Therefore, adult children do not need to provide additional financial support, as the public resources provided by the government, to a certain extent, have replaced the expenditure of family private resources.
(2) Stability of family support funds: Adult children’s financial support for their parents usually depends on the long-term economic situation of the family rather than short-term policy changes, which are relatively rigid. Factors such as family income structure, adult children’s employment and income levels have a more direct impact on the ability to pay support funds. Even if the government increases its attention and improves welfare, it is not enough to significantly change adult children’s financial support behaviour, especially when the family has already reached its spending capacity limit.
(3) The guiding role of policy advocacy and public opinion: The government’s concern for aging is reflected not only in economic investment but also in the promotion of family ethics and social responsibility. Through policy publicity, the promotion of filial piety culture and family care concepts, the government may indirectly encourage children to strengthen their emotional ties with their parents, as evidenced by an increase in the frequency of visits. The promotional effect of such non-material support on the mental health of middle-aged and elderly people should not be overlooked.

### 4.5 Family asset allocation

The impact of government attention on family support is also reflected in asset allocation. The results in columns (1) to (2) of Table 4 show that increased government attention to aging issues significantly increases household savings rates while reduces household debt ratios. This finding indicates that the government improves the financial decision-making behaviour of middle-aged and elderly households by alleviating their economic pressures, thereby enhancing household financial stability and health [63]. Specifically, possible mechanisms include:

**Table 4:** Mechanism analysis: Household asset allocation and lifestyle.

	Household Asset Allocation		Lifestyle		
	Savings (1)	Debt (2)	Social (3)	Recreational Activities (4)	Sleep (5)
Government Attention	0.045***(0.015)	−0.826* (0.459)	0.005*** (0.002)	0.003* (0.001)	−0.007
Control Variables	Yes	Yes	Yes	Yes	Yes
Year FE	Yes	Yes	Yes	Yes	Yes
Household FE	Yes	Yes	Yes	Yes	Yes
City FE	Yes	Yes	Yes	Yes	Yes
Observations	35,637	20,515	35,637	35,637	35,637
Adjusted R-squared	0.830	0.994	0.197	0.313	0.350

*Notes*: All estimates control for survey year fixed effects, household fixed effects, and city fixed effects. * *p <* 0.1, ** *p <* 0.05, *** *p <* 0.01.

(1) Reducing economic uncertainty and risk exposure: Increased government attention is usually accompanied by expanded social welfare, improved medical insurance coverage and public services. These policies effectively alleviate the financial pressure on families in areas such as medical care and elderly care, allowing middle-aged and elderly families to no longer rely on loans or debt to maintain their basic livelihoods, and to have more resources available for savings.
(2) Enhancing economic security and future expectations: Greater government attention helps to enhance the economic security of middle-aged and elderly households, enabling them to hold more optimistic expectations for the future. This confidence encourages households to actively increase their savings in order to cope with potential emergencies or future expenditure needs, thereby achieving a more reasonable asset allocation.

Therefore, the government’s focus on aging issues not only plays a role in mental health, but also has a profound impact on the economic behaviour of families.

### 4.6 Lifestyle

Columns (3) to (5) in Table 4 show the impact of government attention on three indicators of the lifestyle of middle-aged and elderly people: social interaction, cultural and sports activities, and sleep duration. The results show that government attention significantly increases the frequency of social interaction and participation in cultural and sports activities among middle-aged and elderly people, but has no significant impact on their sleep duration.

(1) Expansion of social support networks: The government’s focus on aging has led to the investment and development of more community resources and social platforms, such as gatherings for the elderly, volunteer activities, and interest groups. Policy support has created more social opportunities for middle-aged and elderly people, helping to expand their social support networks, enhance their sense of social belonging, and thereby improve their psychological well-being [64,65].
(2) Positive interaction between physical and mental health: Government policies often encourage older people to participate in various cultural and sports activities, such as fitness, dancing, calligraphy, etc. These activities not only help improve physical health, but also bring spiritual satisfaction, effectively relieving psychological stress and reducing the risk of depression.
(3) The complexity of sleep problems and the marginal effects of policy: Sleep problems among middle-aged and elderly people are often caused by a combination of factors, such as physiological aging, chronic diseases, and changes in lifestyle. These issues require more comprehensive and medically oriented interventions. Although government attention can improve their quality of life and mental state, these interventions have limited impact on sleep disorders rooted in physio-logical factors and therefore do not significantly extend sleep duration.

## 5 Discussion and conclusion

In existing research on middle-aged and older adult mental health, most studies have examined the impact of pension plans [65,66], social networks [67,68], social support [42,44], and air pollution [69,70] from economic, social, and environmental perspectives. However, as government plays an increasingly important role in socioeconomic development, the impact of government actions on middle-aged and older adult mental health cannot be ignored. Although existing literature has examined government action impacts on this population’s mental health, most studies have focused on specific policies, such as long-term care insurance [71,72] and medical insurance [48,73], without considering comprehensive government policy impacts.

In existing research on government attention, most studies have focused on government environmental attention and government digital attention, examining their impacts on corporate green innovation [74], corporate green investment [75], corporate digital transformation [76], and digital economic output efficiency [36]. However, few studies have explored government attention to aging. As China’s aging process accelerates, government attention to aging issues is growing, making aging a perspective that cannot be ignored. Additionally, existing literature has primarily examined government attention impacts on physical health [77,78], neglecting effects on mental health, particularly for vulnerable groups such as the elderly.

Using data from the CHARLS data collected from 2011 to 2020 and municipal government work reports, this study employed text analysis to measure government attention to aging issues and systematically assessed its impact on middle-aged and elderly mental health. Our findings reveal several important insights with significant public health implications.

Our primary finding demonstrates that government attention to aging issues significantly reduces depression among middle-aged and elderly adults, consistent with existing literature showing that increased government attention effectively improves population health outcomes [77–80]. This finding underscores the critical role of government policy prioritization in addressing mental health challenges among aging populations. The mechanism operates through both direct policy implementation and indirect signaling effects that enhance psychological security and social recognition among older adults.

Notably, the impact varies significantly across demographic groups. The positive effects are more pronounced among men and rural populations, suggesting that existing urban-rural disparities in healthcare access may amplify the benefits of government attention in underserved areas. The differential gender effects warrant further investigation, as they may reflect varying social support structures and help-seeking behaviors between men and women.

Environmental quality emerged as a crucial mediating mechanism, with increased government attention significantly reducing wastewater and particulate matter emissions. This finding extends beyond traditional aging policy research by demonstrating how environmental governance can serve as an indirect pathway to improve elderly mental health. Unlike previous studies focusing on environmental governance investments, our research reveals that aging-focused government attention achieves environmental improvements through integrated policy approaches.

The study also reveals that government attention enhances social welfare provision and increases frequency of intergenerational visits, while having minimal impact on direct financial support from adult children. This suggests that government intervention may complement rather than substitute family support systems, particularly in emotional and social domains. Furthermore, government attention significantly improves household financial stability by increasing savings rates and reducing debt ratios, providing economic security that contributes to better mental health outcomes.

Our findings show that government attention promotes participation in social and cultural activities, improving lifestyle factors that support mental health. However, the lack of significant impact on sleep duration suggests that targeted interventions may be needed to address specific health behaviors among older adults.

The practical significance of this paper lies in the following three points: first, this study emphasizes the important practical significance of government attention to aging for improving the mental health of middle-aged and elderly individuals. In previous practice, interventions for mental health issues among middle-aged and elderly people have primarily been proposed from medical, family care, and government policy perspectives. However, there has been relatively limited practice in improving mental health through the indirect effects of government attention to aging. Therefore, this study provides a novel practical approach for alleviating depressive symptoms among middle-aged and elderly populations.

Second, our research findings indicate that government attention to aging has more significant effects on improving mental health among male and rural middle-aged and elderly populations. Consequently, policy implementation should adopt location-specific and targeted approaches, implementing differentiated policies for different regions and population groups to maximize policy effectiveness.

Third, our study reveals that government attention to aging effectively improves the mental health of middle-aged and elderly individuals through four key pathways: environmental improvement, enhanced social welfare, increased frequency of children visiting parents, and expanded community cultural and social activities. Therefore, in policy practice, government attention to aging issues should primarily focus on these four aspects. For example, governments should: (1) significantly strengthen efforts to control water and air pollution; (2) expand the coverage of pension and medical insurance; (3) vigorously promote filial piety culture and traditional values of respecting and caring for the elderly; and (4) actively develop community cultural activity spaces and facilities to help improve the mental health of middle-aged and elderly individuals.

This study is limited by its focus on Chinese contexts and reliance on government work reports as indicators of policy attention. Future research should examine similar relationships in other cultural contexts and explore additional measures of government commitment to aging issues. As populations continue to age globally, this research provides evidence for the public health value of prioritizing aging issues in government policy agendas. The multi-pathway mechanisms identified suggest that comprehensive approaches integrating environmental, social, and economic interventions may be more effective than single-domain policies in promoting healthy aging and mental well-being.

## Supporting information

S1 File Data. (XLSX)
